# Wrist tuberculosis-experience from eighteen cases: a retrospective study

**DOI:** 10.1186/s13018-020-02198-0

**Published:** 2021-01-09

**Authors:** Longfei Zou, Xing Guo, Hao Xue, Denghua Huang, Hui Lv, Meiyun Tan

**Affiliations:** Department of Orthopedic Surgery, The Affiliated Hospital of Southwest Medical University, No. 25 Taiping Road, Luzhou, 646000 Sichuan People’s Republic of China

**Keywords:** Wrist, Tuberculosis, Osteoarticular, Orthopedic procedures, Chemotherapy, Adjuvant

## Abstract

**Background:**

Wrist tuberculosis is a rare disease, which is easy to be misdiagnosed, leading to delayed treatment and poor prognosis. In this study, the clinical manifestations, diagnosis, treatment, and prognosis of 18 cases of wrist tuberculosis were analyzed retrospectively.

**Methods:**

A retrospective study was conducted, investigating tuberculosis of the wrist, diagnosed in 18 patients from August 2013 to November 2018. Puncture biopsy confirmed the diagnosis. The study includes 11 males and 7 females, and 8 left and 10 right wrists. The average age was 53.5 ± 18.3 years and ranged from 15 to 81 years. The disease course was 1 to 42 months, with an average of 15.1 ± 11.3 months. Eighteen patients were underwent surgery and chemotherapy, 3 patients with severe bone defects were treated with wrist fusion, and 15 patients were underwent focus removal. The Gartland and Werley score, DASH score, the range of motion (ROM), grip strength, and imaging examinations were used to evaluate the postoperative recovery of the patients.

**Results:**

Eighteen patients were followed up for 15 to 77 months, with an average follow up of 39.7 ± 15.3 months. The ESR and CRP levels were normal for all patients after chemotherapy. No recurrence of tuberculosis was observed in any of the patients. Among the 15 focus removals, the Gartland and Werley scores at admission, two weeks of chemotherapy, 1 month after surgery, and the last follow-up were 21.73 ± 4.33, 18.60 ± 3.16,11.27 ± 2.79, and 5.07 ± 2.28, respectively; and the DASH scores were 45.87 ± 5.58, 39.47 ± 4.72, 22.67 ± 6.54, and 6.73 ± 2.94, respectively. The range of motion (ROM) of the wrist and grip strength improved significantly when compared to those at admission. Among the three cases of wrist fusion, 2 were fixed with a steel plate and the fixation position of wrist joint was good. One case was fixed with Kirschner wire and resulted in a slightly deformed wrist joint.

**Conclusion:**

For patients with wrist tuberculosis, early diagnosis, preoperative and postoperative chemotherapy, thorough focus removal, and appropriate fixation of the affected limb can help restore the function of the affected wrist, reduce the recurrence rate, and improve the quality of life.

## Background

Tuberculosis is a chronic infectious disease caused by *Mycobacterium tuberculosis*. Tuberculosis infection is still a major health risk in developing countries. Patients infected with the autoimmune deficiency virus and those receiving immunosuppressive therapy are more likely to be infected with tuberculosis than normal individuals [1, 2]. Tuberculosis affects all organs of the human body, and infections of the skeleton are the third most common after tuberculosis and lymphadenopathy, accounting for approximately 10–35% of the impact of tuberculosis outside of the lungs [3]. The spine is the most frequently infected site [4, 5], followed by large load-bearing joints (e.g., hip, knee, and ankle) [6]. Tuberculosis of the joint usually has a long course and is rarely diagnosed before developing arthritis [7]. Although wrist tuberculosis is rare, most of the patients are young and middle-aged. Early diagnosis and treatment may help restore the function of their affected the limb and thereby improve their quality of life. At present, reports on multi-case analysis of wrist tuberculosis are limited, and most articles available are case reports. Kotwal et al. reported 32 cases of the hand and wrist tuberculosis. However, only two patients had a wrist involvement [8]. Benchakroun et al. reported 11 cases of wrist tuberculosis [9]. Until now, no systematic report has been published on a large number of cases of wrist tuberculosis. In addition, there is also no systematic treatment for wrist tuberculosis, and the efficacy is uncertain. The treatment regimen for wrist tuberculosis is also not clear. In other reports of the wrist joints, recurrence is inevitable. In the cases reported by Woon et al., 2 of the 6 patients who were treated with surgery developed recurrent symptoms and underwent a secondary surgery [10]. However, we have collected 18 cases of wrist tuberculosis, which is one of the largest series on wrist tuberculosis. From the patient’s past history, symptoms, physical examination, imaging examination, treatment, and other aspects of analysis were collected. This will contribute to the diagnosis, treatment, and prognosis of similar patients.

## Methods

### Patient data

In this study, we retrospectively analyzed 18 patients with wrist tuberculosis, all of whom visited the Department of Bone and Joint Surgery, Affiliated Hospital of Southwest Medical University from August 2013 to November 2018. These patients fulfilled 3 criteria: (1) preoperative puncture biopsy was diagnosed as a mycobacterium tuberculosis infection of the wrist, (2) no active tuberculosis infection in other parts of the body, and (3) the whole course was followed up. This study included 18 patients (female: 7, male: 11; age: 15–81 years, mean: 53.5 ± 18.3); and 8 patients had tuberculosis of the left wrist and 10 of the right wrist. The course of disease ranged from 1 to 42 months (mean: 15.1 ± 11.3); two patients had a history of pulmonary tuberculosis prior to the onset of carpal tuberculosis. We also reviewed the patients’ history of diseases and identified any systemic diseases or other diseases that may impair the immune system. Preoperative examination revealed that two patients had sinus formation with a purulent exudate in the wrist joint. Before visiting our hospital, five patients had undergone debridement and removal of lesions in other hospitals but did not receive chemotherapy. Physical examination revealed wrist pain, swelling, restricted movement, sinus formation, pus flow, deformity, wrist mass and numbness, pain, stiffness, and mobility disorder; however, no low fever night sweats were observed. General patient information is displayed in Table 1. Blood sedimentation (ESR) was detected by the instrument, normal (0–26 mm/H). Normal values for CRP are 0–5 mg/L.

**Table 1:** Clinical and pathology findings in eighteen patients with tuberculous infection of the wrist

Patient	Age(years)/Sex/Side	Duration (months)	Symptoms	Coexisting disease	Injury factors	Prior wrist surgery	ESR (mm/hour)	CRP (mg/L)	Pathology findings
1	15/M/L	19	Wrist pain, swelling, skin temperature arisen with limitation of motion	Nil	Nil	Nil	62	7.84	Caseating granulomatous with chronic inflammation cells
2	68/M/L	6	Wrist pain, swelling with limitation of motion	Hypertension, coronary heart disease	Toil	Nil	70	16..32	Granulomatous inflammation with Langerhans giant cells
3	50/F/R	12	Wrist swelling and fingers with limitation of motion	Nil	Nil	Nil	119	6.15	Caseating granulomatous, inflammation cells and acid-fast bacilli
4	70/M/R	16	Wrist pain, swelling, purulent secretion, sinus tract and fingers with limitation of motion	Nil	Nil	Nil	83	23.25	Granulomatous inflammation, with Langerhans giant cells
5	49/F/L	1	Wrist pain, swelling, fingers, and wrist with limitation of motion	Nil	Nil	One month ago, the local hospital operated on the wrist	36	1.62	Caseating granulomatous, Inflammation cells
6	58/F/L	30	Wrist pain, swelling with limitation of motion	Nil	Fall on the stairs	One year ago, the local hospital operated on the wrist	38.9	10.25	Chronic inflammation with Langerhans giant cell and rice bodies
7	73/M/R	42	Wrist pain, swelling, skin temperature arisen, and fingers with limitation of motion	Nil	Nil	Nil	30	3.64	Granulomatous inflammation and acid-fast bacilli
8	64/M/R	6	Wrist pain, swelling with limitation of motion and stiffness of fingers	Nil	Nil	Nil	89	15.37	Caseating granulomatous and Langerhans giant cells
9	60/M/R	9	Wrist pain, swelling with numbness of fingers	Nil	Nil	Nil	27	3.99	Inflammation with Langerhans giant cells and acid-fast bacilli
10	55/M/L	8	Wrist pain, swelling with limitation of motion	Nil	Nil	Eight months ago, the local hospital operated on the wrist	32	6.00	Granulomatous inflammation with Langerhans giant cells
11	81/M/R	6	Wrist pain, swelling, malformation, skin temperature arisen, purulent secretion, sinus tract with limitation of motion	Diabetes mellitus	Sprain	Nil	50	146.17	Chronic inflammation cells Acid-fast bacilli and rice bodies
12	41/F/R	24	Wrist pain, swelling, fingers and wrist with limitation of motion, numbness of fingers	Nil	Nil	One year ago, the local hospital operated on the wrist	14	1.12	Caseating granulomatous and inflammation cells
13	69/M/L	15	Wrist pain, swelling with numbness of fingers	Hypertension	Nil	Nil	30	14.26	Caseating granulomatous with Langerhans giant cells and acid-fast bacilli
14	59/F/R	26	Wrist pain, swelling, skin temperature arisen, fingers and wrist with limitation of motion, numbness of fingers	Nil	Fall	Nil	64	38.43	Granulomatous inflammation Inflammation cells and rice bodies
15	18/F/L	5	Wrist pain, swelling with limitation of motion	Nil	Nil	Nil	38	7.35	Granulomatous inflammation Inflammation with Langerhans giant cells
16	60/M/R	1	Wrist pain, swelling with skin temperature arisen	Nil	Nil	Nil	75	74.94	Caseating granulomatous Acid-fast bacilli and rice bodies
17	29/F/R	28	Wrist pain, swelling with limitation of motion	Nil	Nil	Six months ago, the local hospital operated on the wrist	62	8.67	Chronic inflammation with Langerhans giant cell and granulomatous inflammation
18	45/M/L	18	Wrist pain, swelling, malformation with limitation of motion	Hepatitis B	Nil	Nil	58	11.99	Caseating granulomatous Inflammation with Langerhans giant cells and acid-fast bacilli

### Examinations and treatment:

#### Examinations

Preoperative puncture biopsy was used to confirm the diagnosis of wrist tuberculosis, and the puncture was cultured and subjected to a Zieh1-Neelsen stain at the same time. Patients were examined using routine blood, biochemical, and coagulation tests. Radiography and magnetic resonance imaging (MRI) of the affected wrist were used to evaluate the lesion and design the operative procedure. The range of motion (ROM) of the wrist and grip strength was used to evaluate the wrist’s function. The physician-based Gartland–Werley score [11] and the patient-reported DASH (disabilities of the arm, shoulder, and hand) score system [12] were used to indicate the overall functional outcome. Before the culture results, chemotherapy was administered for 2 weeks with four oral anti-tuberculous agents: isoniazid 5 mg/kg, rifampicin 10 mg/kg, pyrazinamide 20 mg/kg, and ethambutol 15 mg/kg per day. After the culture results were obtained, drug administration was adjusted according to the sensitivity of the test results. Cefuroxime was used prophylactically for 3 days during the perioperative period to prevent infection and eliminate bacteria in the joint.

#### Operation

All patients in this study were operated on by the same surgeon. Among the 18 cases, 12 cases were anesthetized using tracheal intubation and 6 cases were anesthetized using brachial plexus. All patients were placed in the supine position, and a balloon tourniquet was bound to the upper arm to prevent intraoperative hemorrhage. The tourniquet was inflated to 200 KPa. A 6 cm S-shaped incision was made on the affected side through the wrist. The skin and subcutaneous tissue were cut layer by layer. For lesions of the wrist joint on the palm side, the tendon of the palmaris longus muscle was pulled to the radial side, the deep fascia and transverse carpal ligament were opened, the median nerve was carefully dissociated, and the tendon of the wrist was separated from the supporting band of the flexor carpi. Numerous rice bodies and gray caseous substances were observed in and around the wrist for four patients. The lesion was curetted repeatedly to remove all the rice bodies, caseous substance, and surrounding necrotic tissue. The removed tissues were sent for pathological biopsy, general bacterial culture, bacterial and fungal smear, and acid-fast staining tests. In some patients, severe erosion of the carpal bones was found intraoperatively, and the residual bone mass was considered inadequate for stabilizing the wrist and restoring its function after removal of the necrotic bone. The right length and shape of the steel plate were chosen, in line with the functional position of the wrist joint. The proximal end of the plate was fixed to the radius, the distal end of the plate was fixed to the third metacarpal, and the wrist joint was fixed to the functional position. If the Kirschner wire would fix the wrist firmly, a 2.0-mm Kirschner wire was inserted into the ulnar and radial sides to fix the wrist in the functional position. The incision was repeatedly rinsed with normal saline, hydrogen peroxide, and iodophor. After exploring the carpal canal to ensure the absence of evident compression, two rubber drainage strips were placed and the wound was wrapped with a sterile dressing. Except for those treated by plating, all patients were fixed with plaster and the carpal joint was adjusted to the functional position.

#### Postoperative treatment

After surgery, all patients were administered intensive treatment with isoniazid (5 mg/kg), rifampicin (10 mg/kg), pyrazinamide (20 mg/kg), and ethambutol (15 mg/kg) tetrad anti-TB drugs for 3 months, followed by the oral drugs isoniazid (5 mg/kg) and rifampicin (10 mg/kg) for 9 months. Liver and kidney functions were reviewed every month. Patients were followed-up every 4 weeks for 6 months after discharge. Patients were then followed-up every 8 weeks from 6 months to 1 year. In the second year, patients were followed-up every 3 months for the first 6 months and then every 6 months. During each follow-up, ESR and CRP were measured until they returned to normal levels. X-rays were used to assess wrist recovery at each follow-up.

#### Outcome measurement

Patients were followed-up by the Gartland and Werley scale and the DASH score, and wrist function was evaluated using the ROM and grip strength. Wrist mobility (flexion, extension, supination, and pronation) was measured with a goniometer and grip strength with a Jamar dynamometer. The Gartland-Werley scale is a physician-based scoring system that includes residual deformity, subjective findings, ROM, postoperative complications, and poor finger function. For scores ranging from 0 to 52, a higher score indicated a worse wrist function. DASH is a validated patient report questionnaire for assessing the patient’s ability to perform daily activities. For scores ranging from 0 to 100, 0 indicated no disability and 100 indicated maximum disability. If a patient experienced increased pain or other symptoms of discomfort during outpatient follow-up, further examinations, including CT scan or MRI, were considered, both of which were performed in the outpatient clinic.

#### Statistical analysis

All statistical analyses were conducted using SPSS Statistical Software (SPSS for Windows, version 23.0). Data are presented as the mean and standard deviation. ANOVA was used to compare the differences between Gartland and Werley’s wrist score and DASH. *P* values less than 0.05 were considered statistically significant.

## Results

A total of 18 patients were followed-up for 15 to 77 months with an average of 39.7 ± 15.3 months. There were two cases of the peripheral skin defect, one of which healed after local dressing and the other recovered after a skin graft. All patients completed chemotherapy treatment under direct observation therapy (DOT). There were no adverse reactions nor changes to the chemotherapy regimens. The culture of wrist secretions did not produce any drug-resistant bacteria. No patients showed any recurrence symptoms during the follow-up of at least 15 months, the ESR and CRP levels were normal for all patients after chemotherapy, and X-ray examination showed no lesions in the affected side of the wrist. For the three patients with a wrist joint fusion, for two of them internal fixation with a steel plate was employed. During the follow-up, the steel plate was confirmed to be not loose, fractured, or displaced. One patient was fixed with a Kirschner wire. During the 8 months of follow-up, Kirschner wire displacement was performed. Finally, the palm deviated to the ulnar side. Gartland and Werley and DASH scores of the 15 patients that underwent simple lesion removal were statistically analyzed during admission, 2 weeks after chemotherapy, 1 month after surgery, and at the last follow-up. The difference was statistically significant **(**Table 2**)**. The ROM and grip strength significantly improved (Table 3).

**Table 2:** Gartland-Werley and DASH score on admission, 2 weeks of chemotherapy, 1 month after surgery and last follow-up

Point-in-time	Gartland–Werley score (points)	DASH (points)
On admission	21.73 ± 4.33	45.87 ± 5.58
Two weeks of chemotherapy	18.60 ± 3.16	39.47 ± 4.72
One month after surgery	11.27 ± 2.79	22.67 ± 6.54
Last follow-up	5.07 ± 2.28	6.73 ± 2.94
F	72.228	483.182
P	<0.0001	< 0.0001

**Table 3:** Comparison of ROM and grip strength of the operated wrist on admission, 2 weeks of chemotherapy, 1 month after surgery and last follow-up

	On admission		Two weeks of chemotherapy		One month after surgery		Last follow-up	
	Mean(SD)	% of value on contralat. side	Mean(SD)	% of value on contralat. side	Mean(SD)	% of value on contralat. side	Mean(SD)	% of value on contralat. side
Flexion (°)	43.3 (6.6)	65.7	47.7 (5.6)	70.3	53.7 (5.1)	82.7	61.9 (5.0)	95.5
Extension (°)	44.0 (7.3)	62.4	48.5 (5.8)	68.3	53.7 (5.8)	78.4	62.9 (4.7)	91.9
Pronation (°)	59.0 (8.7)	67.5	63.5 (7.3)	72.1	68.1 (6.7)	76.3	77.3 (4.6)	87.6
Supination (°)	56.1 (5.3)	58.7	61.7 (5.6)	62.3	67.7 (5.2)	73.9	75.9 (4.8)	81.4
Radial deviation (°)	13.8 (4.0)	63.0	15.8 (3.3)	67.6	17.7 (2.9)	81.3	21.4 (3.2)	93.0
Ulnar deviation (°)	24.3 (4.3)	73.3	25.7 (4.4)	76.9	28.1 (3.5)	85.5	32.1 (2.6)	92.2
Grip strength (kg)	12.7 (5.6)	43.3	15.2 (4.7)	50.5	20.5 (3.5)	68.3	28.1 (4.7)	93.5

### Selected case reports

**Fig. 1:**
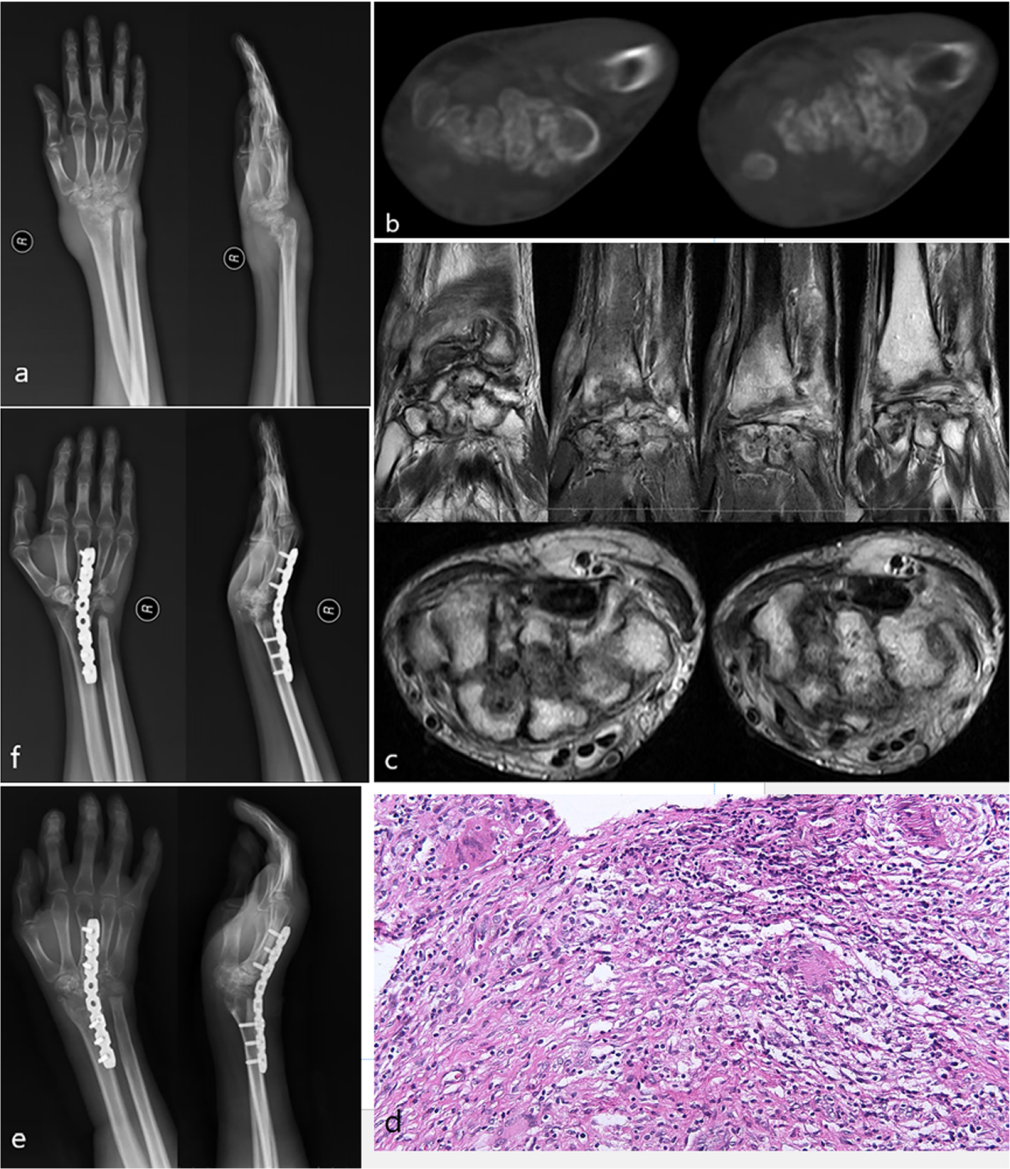
**a** Conventional radiography anterior posterior and lateral view pre-operation, **b** CT scan pre-operation, **c** pre-operative MR images, **d** the biopsy pre-operation, **e** X-ray scan 1 month post-operation, **f** X-ray scan 48 months post-operation

A 29-year-old right-handed woman had no other complications and no previous history of tuberculosis. The symptoms were progressive pain, swelling, and limited movement of the right wrist for 24 months. Physical examination showed that the skin temperature of the right wrist was increased and that there was a 4-cm mass on the dorsal side, which had wave motion; however, the skin was not broken. Wrist mobility was limited and the range of activity was recorded. An *X*-ray of the wrist showed the collapse of the joint (Fig. 1a). A CT of the wrist showed extensive damage to the distal radius and carpus, disordered bone structure, increased patchy density shadow, and multiple gritty changes (Fig. 1b). An MRI of the wrist showed that the structure of the wrist joint was disordered, the carpal bone was damaged, the surrounding soft tissue was swollen, and that multiple tendons were damaged (Fig. 1c). The results of the biopsy showed Langerhans giant cells and chronic granulomatous inflammation in the right wrist (Fig. 1d). Zieh1-Neelsen staining was positive. The ESR level was 62 mm/h, whereas the CRP level was 8.67 mg/L. After receiving rifampicin, isoniazid, pyrazinamide, and ethambutol for 2 weeks, surgical treatment was performed, and the pathological synovial tissue was resected and internal fixation performed. Resection of the lunate and capitate bone was carried out during the operation. Histology showed necrotizing granulomatous inflammation. An 8-hole 2.7-mm stainless steel plate was used to fix the joint. The proximal part was fixed to the radius, and the distal part was fixed to the third metacarpal. The established chemotherapy plan was strictly followed after the operation. The incision healed completely at 2 weeks, and no recurrence was observed at 48 months of follow-up.

## Discussion

Wrist tuberculosis is rare in all bone tuberculosis, accounting for less than 1% of all cases of bone tuberculosis [13]. Early diagnosis of this disease is difficult, and therefore, the misdiagnosis rate is quite high. It takes an average of 16–19 months from the onset of carpal symptoms to a clear diagnosis [14]. Subtle clinical and unremarkable radiographic features are the main reasons for a delayed diagnosis of TB [15]. In our patients, the course of the disease was 15.1 ± 11.3 months. Tuberculosis of the wrist can originate from trauma-related infection or through the pathogen transport by blood circulation from the other organs. In our study, four (22%) patients had a history of wrist trauma. The diagnosis of overuse injury was incorrect, and TB could have been suspected on the basis of persistent low grade pyrexia and fatigue [16]. Bone tuberculosis is often metastasized from tuberculosis elsewhere, but in this group, only 2 patients (11%) had prior tuberculosis. Benchakroun et al. described 11 cases of wrist tuberculosis, of which 4 patients were or are currently suffering from tuberculosis [9]. Kotwal reported 32 cases of wrist and hand tuberculosis with no active tuberculosis elsewhere in the body [8]. Pain and swelling are the most common symptoms of wrist tuberculosis [17]. In this group of cases, all patients had varying degrees of wrist pain and swelling. However, only two patients had sinus tract formation. In contrast, Prakash reported 44 cases of wrist and hand tuberculosis in children, and cutaneous sinus tract was present in 31% of the patients. Thus, sinus tract formation is more common in wrist and hand tuberculosis in children [18]. In our case, granulomas in the wrist were present in almost all patients. However, only 4 patients had melon seeds “or” rice bodies at the wrist. However, a melon seeds “or” rice bodies appeared in 50% of the cases reported by Aboudola et al. [19]. In all 18 patients, preoperative chemotherapy was followed by surgery 2 weeks later, and postoperative chemotherapy was continued. None of the patients exhibited recurrent symptoms during at least 15 months of follow-up. However, in the study reported by Woon et al., 2 of the 6 patients treated with surgery developed recurrent symptoms and a secondary surgery was required. We believe that this chemotherapy regimen combined with surgery is a better method for the treatment of wrist tuberculosis. All patients completed their chemotherapy under direct observation therapy (DOT). This is considered a factor in our treatment success. The follow-up results showed that Gartland, Werley, and DASH scores of all patients were significantly reduced, and wrist ROM and grip strength were significantly improved.

**Fig. 2:**
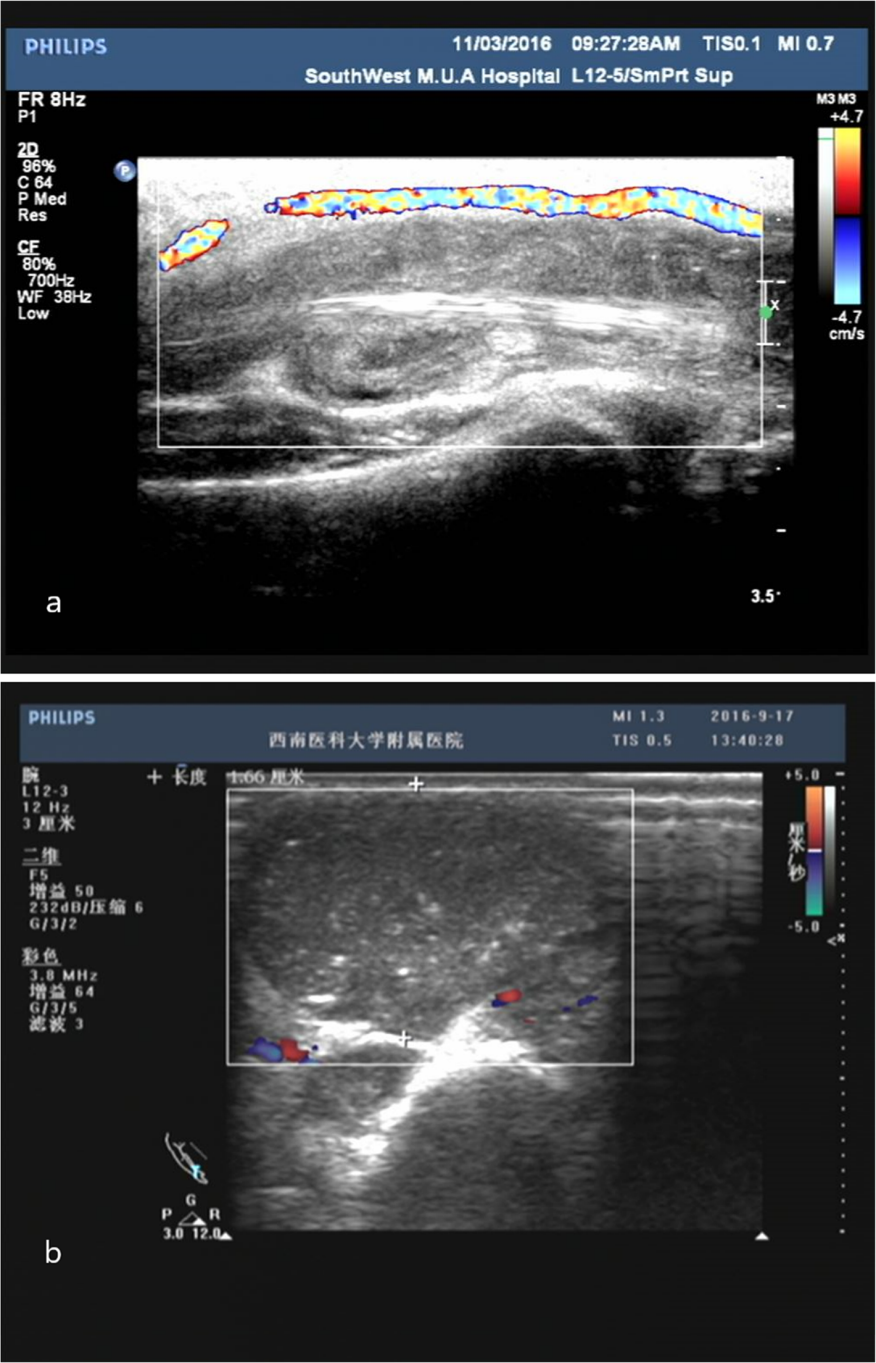
**a**, **b** Ultrasonography showing a cystic mass of the wrist with clear boundaries and an irregular shape, in which hyperechoic echoes can be seen

Blood tests of patients with wrist tuberculosis revealed increased levels of ESR and CRP and decreased levels of albumin. Radiographically, most the lesions showed wrist masses with unclear boundaries, bone destruction, narrowing of the carpal joint space, and involvement of the distal radius and ulna. With disease progression, the invasion of the carpal bones by the lesion became more advanced, forming extensive periostitis [20]. However, in the case of simple synovial tuberculosis, there was no clear radiographical sign of osteoporosis or bone destruction. During the early stage of wrist tuberculosis, it is difficult to radiographically diagnose the lesion. In case of evident radiographical signs of joint destruction and calcification, further imaging examinations are warranted [21]. MRI is an effective tool for identifying carpal tuberculosis [22], and can clearly show the soft tissue of the wrist, thereby offering a clearer understanding of the tendon injury, blood vessel, and articular cartilage. It can also detect early effusion in the wrist as well as the inflammatory changes of bone. The carpal tuberculosis MRI showed rice bodies in the carpal mass and abnormal thickening of the synovium. On T2WI, the low-density lesions, low-density synovium with central erosion, and surrounding abscess are important markers that distinguish tuberculosis from other types of arthritis [23, 24]. MRI examination allows for an early diagnosis of wrist tuberculosis. In recent years, color Doppler ultrasonography had also commonly been used for the diagnosis of wrist diseases (Fig. 2). For example, during effusion and empyema in the wrist, ultrasonography can clearly identify their location and volume and reveal the involvement of the blood vessels, nerves, and tendons in the wrist joint [21]. Histopathology is the gold standard for the diagnosis of tuberculosis [25].

**Fig. 3:**
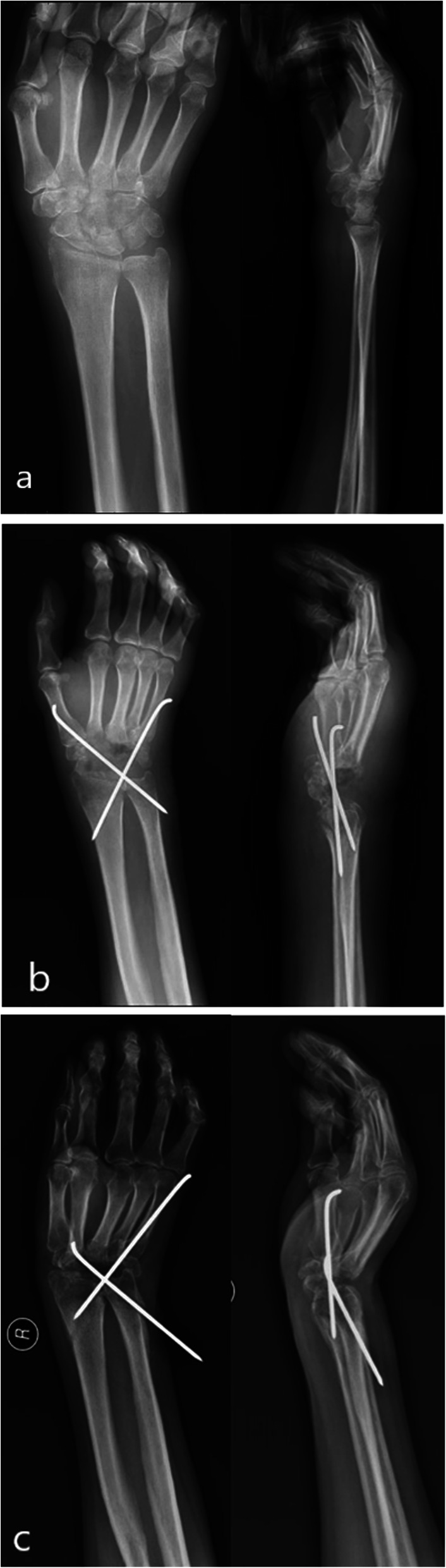
**a** Conventional radiography anterior posterior and lateral view pre-operation; **b** X-ray scan 1 month post-operation, the position of the Kirschner wire is not shifted; **c** X-ray scan 8 months post-operation, the position of the Kirschner wire is significantly displaced and the wrist is fused in a position towards the ulnar deviation region

Early use of anti-tuberculosis drugs is key to successful treatment [18]. In our study, five patients received antecedent surgical clearance of the focus of the wrist. However, they were not given anti-tuberculous postoperatively, a factor that likely contributed to the recurrence of wrist tuberculosis. Surgical debridement without prescription of chemotherapeutic agents may lead to the recurrence of wrist tuberculosis [26]. During operation, it is advisable not to excessively pursue a small incision. Instead, it is essential to fully expose the focus, protecting the blood vessels and nerves of the wrist, and thoroughly remove the focus tissue, especially the caseous substance and rice bodies, including the necrotic carpal bone, tendon, tendon sheath, and the surrounding soft tissue, and to fully scrape the focus tissue with a curette until the normal bone surface and tissue ooze blood. In case of a severely erosion of the carpal bones, the wrist function of cannot be restored after removing the necrotic bone, joint fusion can be performed. Arthrodesis can be performed with steel plates or Kirschner wires. The steel plate should be used for internal fixation if there are more necrotic carpal bones and if the wrist joint is unstable after more carpal bones are removed. Compared to the Kirschner wire, the steel plate fixation is more reliable; however, the cost is higher. If there is a small amount of wrist bone removal and if the Kirschner wire can be fixed firmly, the patient and their guardian should be consulted. A Kirschner wire fixation may fix the wrist in a deformed position. Among the three cases of wrist fusion, 2 were fixed with a steel plate and the fixation position of wrist joint was good. One case was fixed with a Kirschner wire and resulted in a slightly deformed wrist joint (Fig. 3).

## Conclusions

For patients with wrist tuberculosis, early diagnosis, preoperative and postoperative chemotherapy, thorough focus removal, and appropriate fixation of the affected limb can help restore the function of the affected wrist, reduce the recurrence rate, and improve the quality of life.

## Data Availability

The datasets used and/or analyzed during the current study available from the first author on reasonable request.

## Abbreviations

DASH: Disabilities of the arm, shoulder, and hand
ROM: Range of motion
ESR: Erythrocyte sedimentation rate
CRP: C-reactive protein
CT: Computed tomography
MRI: Magnetic resonance imaging
CDFI: Color Doppler flow imaging
DOT: Direct observation therapy
